# 

**Published:** 1888-02

**Authors:** 


### CONTENTS.

### COMMUNICATIONS.

Five Reasons for Using Heavy Gold Foil in Filling Teeth.............. 57
Medical Treatment Pior to, or in Connection with, Filling Teeth...... 59

### PROCEEDINGS.

Southern Dental Association.......................................... 63
Ninth International Medical Congress.................................. 84

### SELECTIONS.

Abscess of the Lung, Probably Caused by the Stump of a Tooth Passing into Right Bronchus........................................ 92
For Chapped Hands.................................................... 94
Elegant Mouth Wash................................................... 94
The Part Played by the Nose and the Anterior Respiratory Passages in Respiration....................................................... 95
Learning to Use Artificial Teeth..................................... 96
Fatal Result of Cigarette Smoking.................................... 97
A Secret of Success.................................................. 97
The Treatment of Facial Neuralgia by Antipyrine...................... 98
The Legal Status of Crown and Bridge Work............................ 99
Salmagundi........................................................... 102

### CORRESPONDENCE.

A New Tooth Crown.................................................... 105
Don't Forget the Mississippi Valley Association of Dental Surgeons 107
Obituary............................................................. 108

